# Multiparametric mri-based radiomics nomogram for predicting lymph-vascular space invasion in cervical cancer

**DOI:** 10.1186/s12880-024-01344-y

**Published:** 2024-07-05

**Authors:** Feng-Hai Liu, Xin-Ru Zhao, Xiao-Ling Zhang, Meng Zhao, Shan Lu

**Affiliations:** 1 Department of Magnetic Resonance Imaging, Cangzhou Central Hospital, No. 16, Xinhua West Road, Cangzhou City, Hebei Province 061001 China; 2 Department of Pathology, Cangzhou Central Hospital, Cangzhou City, 061001 Hebei Province China

**Keywords:** Uterine cervical neoplasms, Radiomics, Lymph-vascular space invasion, Magnetic resonance imaging

## Abstract

**Purpose:**

To develop and validate a multiparametric magnetic resonance imaging (mpMRI)-based radiomics model for predicting lymph-vascular space invasion (LVSI) of cervical cancer (CC).

**Methods:**

The data of 177 CC patients were retrospectively collected and randomly divided into the training cohort (*n*=123) and testing cohort (*n* = 54). All patients received preoperative MRI. Feature selection and radiomics model construction were performed using max-relevance and min-redundancy (mRMR) and the least absolute shrinkage and selection operator (LASSO) on the training cohort. The models were established based on the extracted features. The optimal model was selected and combined with clinical independent risk factors to establish the radiomics fusion model and the nomogram. The diagnostic performance of the model was assessed by the area under the curve.

**Results:**

Feature selection extracted the thirteen most important features for model construction. These radiomics features and one clinical characteristic were selected showed favorable discrimination between LVSI and non-LVSI groups. The AUCs of the radiomics nomogram and the mpMRI radiomics model were 0.838 and 0.835 in the training cohort, and 0.837 and 0.817 in the testing cohort.

**Conclusion:**

The nomogram model based on mpMRI radiomics has high diagnostic performance for preoperative prediction of LVSI in patients with CC.

**Supplementary Information:**

The online version contains supplementary material available at 10.1186/s12880-024-01344-y.

## What is already known on this topic?

1. Cervical cancer is one of the leading causes of cancer-related death in women.
2. Lymph-vascular space invasion (LVSI) is a highly risk factor for tumor recurrence, and it is difficult for radiologists to diagnose LVSI macroscopically.
3. The rapid development of radiomics has made it possible to predict LVSI in cervical cancer.

## What this study adds?

1. In this paper, we developed an mpMRI-based radiomics model that predicts LVSI in cervical cancer by combining features extracted by multiparametric imaging techniques, including T2WI, DWI, and DCE-T1WI sequences.
2. We selected 13 of the most important features from a large number of imaging features and constructed a rad-score accordingly for assessing the risk of LVSI in patients.
3. The model showed good diagnostic performance in both training and test cohorts.

## How this study might affect research, practice or policy?

1. This approach takes advantage of radiomics to transform medical images into high-dimensional data from which quantitative imaging features are extracted to aid clinical decision-making.
2. This model has high accuracy and clinical application value in predicting LVSI.
3. This study provides a potentially non-invasive tool for preoperative prediction of LVSI in patients with cervical cancer, which has important clinical implications in the diagnosis and treatment decision-making of cervical cancer.

## Introduction

Cervical cancer (CC) is one of the leading causes of cancer-related death in women [1, 2]. One of the key steps in tumor cell spread is lymph-vascular space invasion (LVSI). LVSI increases the risk of metastasis in cancer cells [3–5]. Although vascular space invasion (VSI) has no significant effect on CC staging, it can affect the treatment approach [6]. Meanwhile, LVSI can significantly reduce the 5-year survival rate of CC patients [7, 8].

However, due to the limited ability of traditional examination to evaluate LVSI, it is difficult for radiologists to diagnose LVSI with the naked eye. And preoperative needle biopsy and conization specimens are also difficult to diagnose LVSI [9]. At present, the diagnosis can only be made by postoperative pathological evaluation. Magnetic resonance imaging (MRI) has the advantages of multi-parameter, multi-directional imaging, and high soft tissue resolution, and has been an essential part of diagnosing and staging CC. But it still cannot provide intuitive information to identify LVSI.

Radiomics is an advanced medical technology that converts these features into high-dimensional datasets by extracting a large number of quantitative features from medical images and uses these data for in-depth analysis to assist clinical decision-making. Significant advances have been made in radiomics over the last decade, particularly in predicting LVSI in CC [9–11]. To further improve the precision of CC treatment, the aim of this study was to develop and validate an MR-based radiomics scoring tool. In this study, not only the key features of T2WI, DWI, and DCE-T1WI radiomics were combined, but also the clinical independent risk factors were combined to construct a fusion model and nomogram, which greatly improved the accuracy and clinical application value of predicting LVSI. Through this study, we expect to provide clinicians with a more precise and practical tool to help them make more informed decisions in the treatment of CC, thereby improving the treatment outcome and quality of life of patients.

## Materials and methods

### Patients

**Fig. 1:**
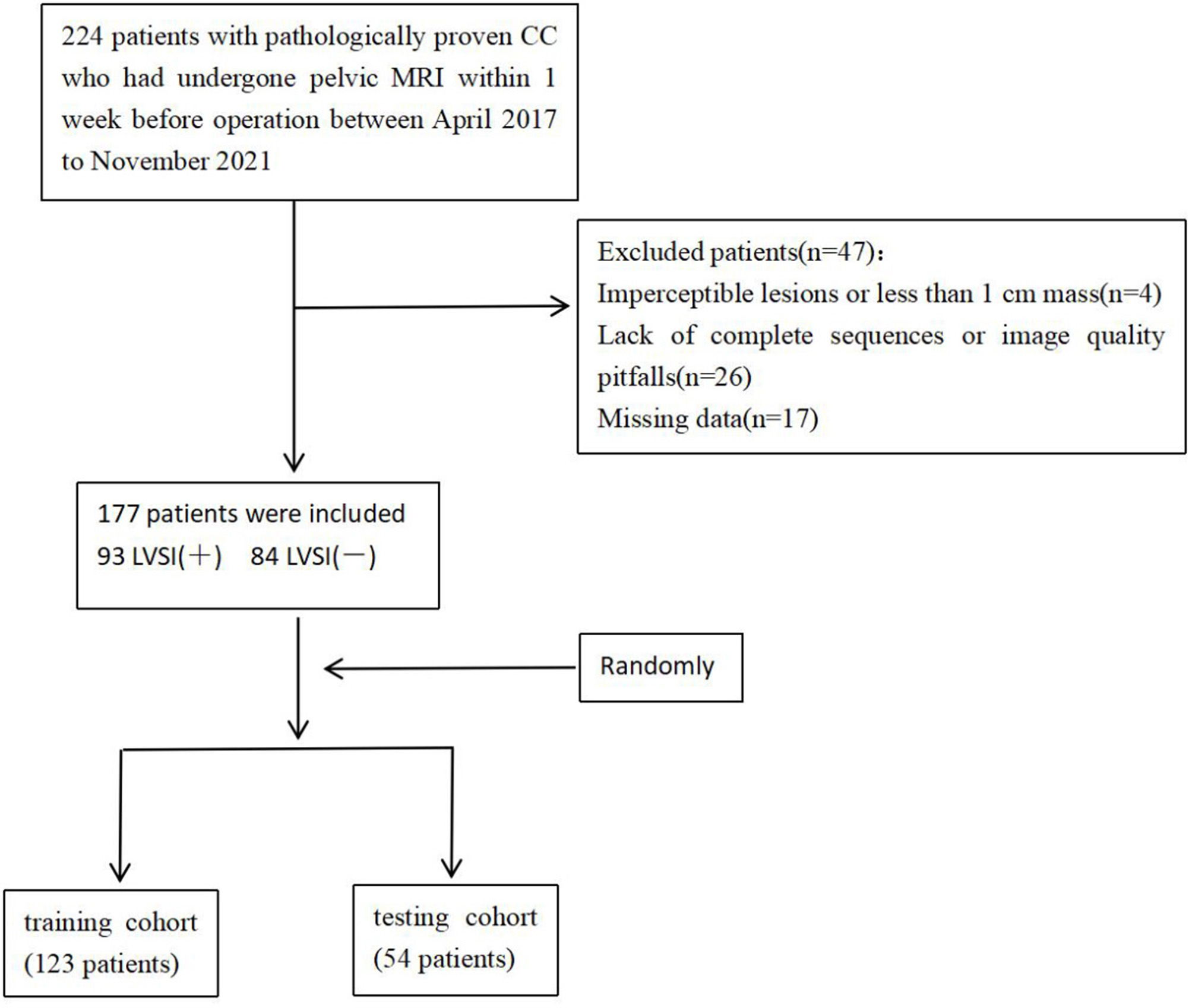
Flow diagram shows inclusion and exclusion criteria

From April 2017 to November 2021, 177 patients with pathologically proven CC and preoperative pelvic MRI were retrospectively chosen. Inclusion criteria for this study were as follows: 1) the MRIs were performed within 1 week before operation 2) no history of treatment for CC before MRI; 3) CC was diagnosed according to the ' Guidelines for the Diagnosis and Treatment of CC and confirmed by pathology, suggesting LVSI status [12]. The exclusion criteria were: 1)image quality of the main sequences not suitable for drawing an explicit volume of interest (VOI) ; 2) no lesion perceptible on MRI or the mass less than 1 cm; 3) incomplete clinical data. Finally, 177 patients were included in this study and subdivided into a training cohort (123 patients) and a testing cohort (54 patients) with a ratio of 0.7:0.3 randomly (Fig. 1). In training and test cohort, the number of LVSI (+) patients was 65 and 28, and the number of LVSI (-) patients was 58 and 26, respectively.

All the procedures performed in studies involving human participants were in accordance with the ethical standards of the institutional and/or national research committee and with the 1964 Helsinki Declaration and its later amendments or comparable ethical standards. This retrospective study was approved by the institution’s Committee on Human Research (ethical batch number: 2014-003-01), and the need for obtaining written informed consent was waived.

### MRI protocol

MRIs were performed with a 3.0T imaging unit (GE Healthcare, Milwakee, WI, USA) with a phased-array 8-channel sensitivity encoding abdominal coil. Anisodamine (except for contraindications) 10 mg was intramuscularly injected 20 min before scanning to inhibit peristalsis of bowel. The set image protocol included a small field-of-view(sFOV) high-resolution(HR)-T2-weighted MRI (T2WI), diffusion-weighted imaging (DWI) and dynamic contrast-enhanced-T1-weighted imaging(DCE-T1WI). Each patient was administered an intravenous injection of 0.1 mmol/kg of Gadoteric Acid Meglumine Salt Injection. Seven scanning images were obtained with a total scanning time of 140s. The images in the last scanning period (DCE-T1WI-equilibrium phase) were selected for target delineation. The parameters of the three sequences are listed in Table 1.

**Table 1:** MRI protocols

Sequences Parameters	T2WI	DWI	DCE
Acquisition plane	Axial oblique	Axial	Axial
TR (ms)	4395	4800	4.3
TE (ms)	102	100	2.0
Slice thickness (mm)	3.0	7.0	4.0
Acquisition matrix	320 × 288	128 × 128	256 × 192
FOV (mm)	200 × 200	420 × 336	420 × 336
Interslice gap	0.5	1.0	0

Note DCE: Dynamic contrast-enhanceed; TR: Repetition time; TE: Echo time; FOV: Field of view. The axial oblique plane was perpendicular to the long axis of the uterus

### Tumor segmentation and radiomic features extraction

**Fig. 2:**
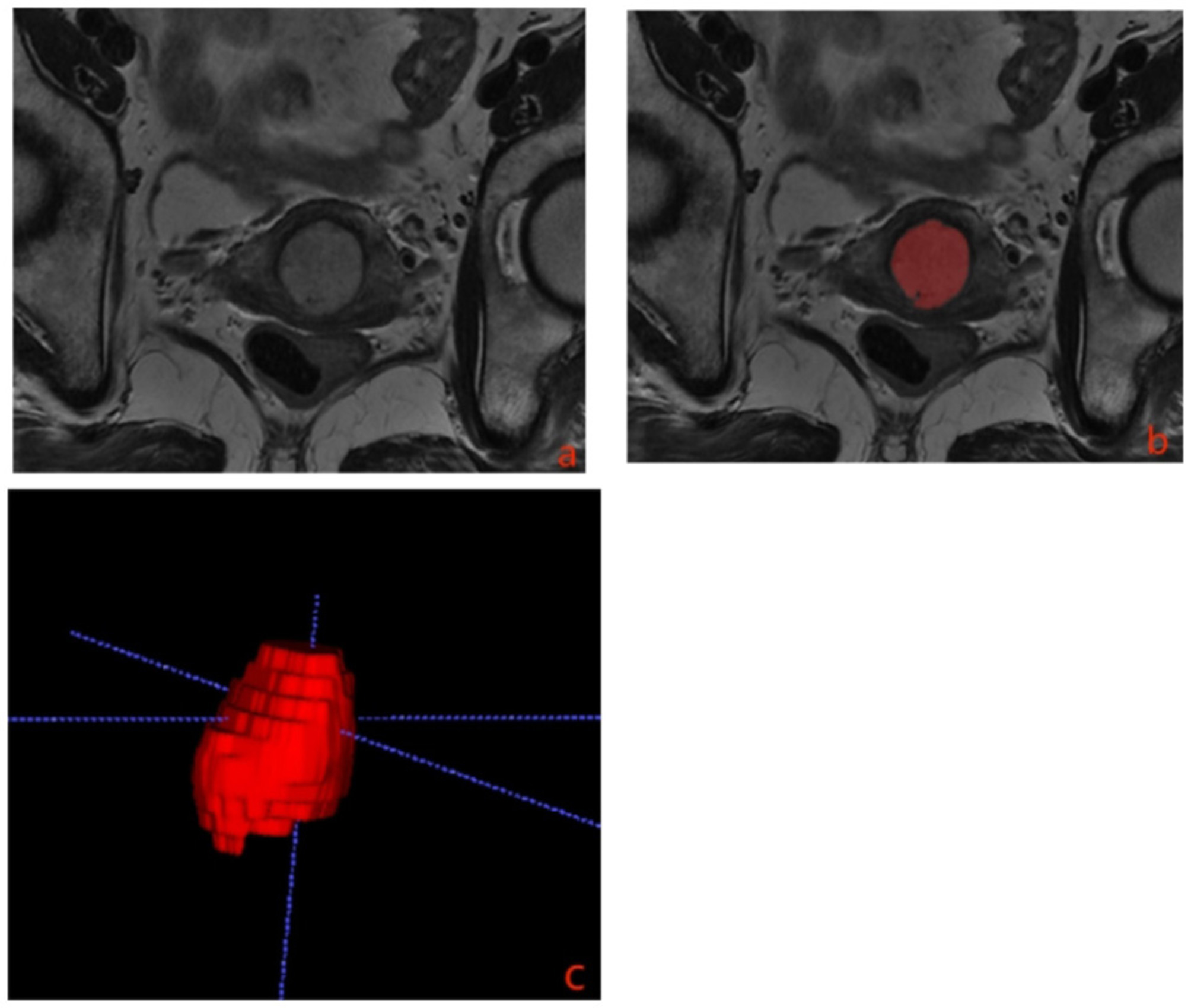
**a** Axial oblique T2WI images of a patient with cervical cancer. **b** Using all image slices in which the tumor appears, the segmentation is done manually along the whole lesion margin. Regions of interests (ROI) of the tumor lesions were manually delineated. **c** Three-dimensional volumes of interest (3D-VOIs)

Digital Imaging and Communications in Medicine (DICOM) images including T2WI, DWI, and DCE-T1WI-equilibrium phase maps were downloaded from our picture archive and communication system (PACS) and transferred to the ITK-SNAP (v3.6.0, http://www.itksnap.org) for segmentation of manual MR images. These images were taken from a single institution. For evaluation of the reliability of the radiomics features, three-dimensional volumes of interest(3D-VOIs) of tumors were strictly segmented by the two radiologists with more than 5 years of experience in magnetic resonance diagnosis, respectively. Each radiologist independently annotated the same patient. ICC analysis was used to assess the consistency of ROIs between the two radiologists. By ICC analysis, the reliability coefficient was > 0.75, indicating good reliability and high consistency of ROIs. A third radiologist will be evaluated if there is a large difference between the two. Given the importance of heterogeneity analysis, the regions of interest (ROIs) should include necrosis and cystic tissue(Fig. 2). Multiple sequences are cross-referenced when the tumor boundary is unclear to minimize delineation bias. Before feature extraction, MRI images were standardized, including image interpolation, intensity normalization, and gray-level discretization. The parameters used for the three standardizations were as follows: Interpolation method: Cubic spline interpolation was used. Intensity normalization method: Select to map the signal range between 0 and 1. Gray-level discretization: 32 Gy-levels are selected according to the dynamic range and resolution of the image. Radiomic features were extracted from ROIs with the PyRadiomics software, including first-order features, shape-based features, and texture features. The texture features were computed from gray-level co-occurrence matrix(GLCM), gray-level run-length matrix(GLRLM), gray-level size zone matrix(GLSZM) and gray-level dependence matrix(GLDM).

### Features selection and radiomics models construction

**Fig. 3:**
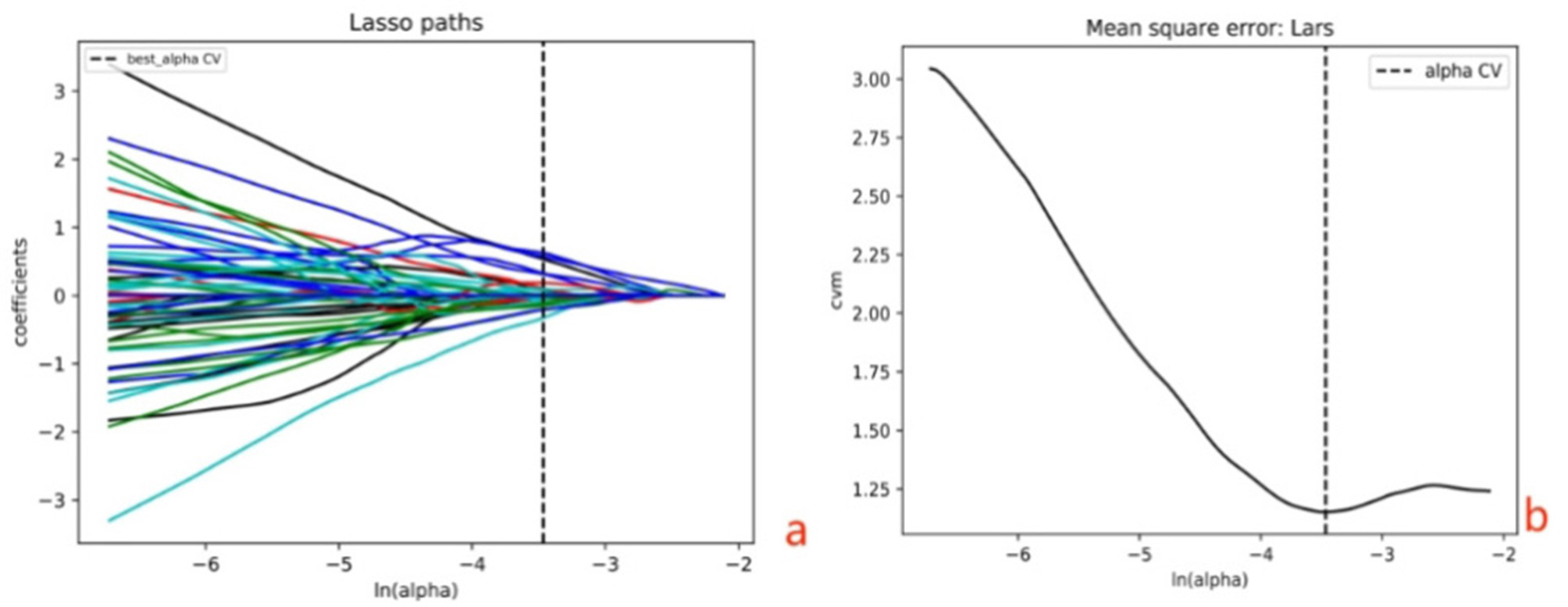
LASSO regression model and 10-fold crossover regression were used to extract features. **a** Coefficient Convergence Plot; **b** The minimization criterion is used to obtain the optimal log(λ) value with the smallest binomial deviation

In order to improve the accuracy of radiomics model in predicting LVSI in CC, we adopted a series of complex data processing and feature selection steps. First, we use the Minimum Redundancy Maximum Correlation (mRMR) algorithm to screen the initial feature set to eliminate those features that are repetitive or have low association with the target variable. Next, we applied the least absolute shrinkage and selection operator (LASSO) method to reduce the features and identified significant coefficients on the training set (see Fig. 3). The base model for predicting LVSI status was logistic regression.

Based on these selected features and their correlation coefficients, we developed a scoring system for assessing each patient ‘s radiomics profile, called Rad-score. To validate the predictive ability of the model, we constructed three independent models based on three different sequences of magnetic resonance imaging (MR): T2-weighted imaging (T2WI), diffusion-weighted imaging (DWI), and dynamic contrast-enhanced T1-weighted imaging (DCE-T1WI) in equilibrium phase. In addition, we created a comprehensive multiparametric magnetic resonance imaging (mpMRI) radiomics model by weighting the main features of these three sequences and their corresponding coefficients. The multiparametric features are to combine the features of multiple single sequences to obtain a new feature set. We evaluated the diagnostic efficacy of these models by receiver operating characteristic (ROC) curves and picked out the best performing mpMRI radiomics model from them. Based on this optimal model, we further constructed a nomogram designed to provide clinicians with an intuitive, easy-to-use tool to assist them in assessing the risk of lymph node metastasis in patients with CC.

### Radiomics nomogram construction

Univariate logistic regression analysis was used for age, histology type, FIGO stage and differentiation degree. FIGO stage is based on imaging. The above parameter information was determined by imaging and needle biopsy before performing the procedure for nomogram construction. A nomogram based on the Radscore and selected clinical parameters was built into the training cohort to predict the potential for LVSI of CC. ROC curve analysis was performed to illustrate the model performance. Meanwhile, we calculated the area under the curve (AUC), specificity, sensitivity, and accuracy in further evaluating the MRI models and radiomics nomogram performance. A calibration curve was applied to evaluate the probabilistic prediction performance of the nomogram. Decision Curve analysis (DCA) was used to calculate the net benefit within the probability threshold to evaluate its clinical application value.

### Pathological examination

All patients included in this study received radical hysterectomy. Pathological indicators included tumor grade, histology type and presence of LVSI. The pathological results were obtained by a pathologist with over 5-year experience and a pathologist with over 10-year experience.

### Statistical analysis

SPSS 26.0 software was used for statistical analysis. The quantitative data were described by ‾x ± s, and the t test was used for comparison between groups. Chi-square test or Fisher’s exact test was used for comparison between groups. The larger the area under the curve (AUC), the higher the diagnostic accuracy. The DCA curve is a simple method for evaluating clinical predictive models, diagnostic tests, and molecular markers. p-value < 0.05 was considered statistically significant.

## Results

### Baseline characteristics of patients

A total of 177 female patients, aged from 24 to 80 years, with an average age of 52.16 ± 10.29 years, were included in the study based on the inclusion and exclusion criteria. There were 93 cases in LVSI positive group and 84 in LVSI negative group. There were no significant differences in age, histology type, and differentiation degree between the two groups, while the FIGO stage showed a statistical difference between patients. Patient characteristics are summarized in Table 2.

**Table 2:** Clinicopathological characteristics of patients

Characteristics	LVSI(-) (*n* = 93)	LVSI(-) (*n* = 84)	t/χ2	*P*
**Age**	**51.31 ± 10.22**	**53.1 ± 10.35**	−1.152	0.251
**Histology**			2.251	0.324
SCC	80	71		
AC	13	11		
non-SCC or AC	0	2		
**Grade**			0.868	0.648
Low grade	29	21		
Moderate grade	52	52		
High grade	12	11		
**FIGO stage**			10.181	0.017
I	27	34		
II	55	48		
III	11	1		
IV	0	1		

Note: LVSI: lymph-vascular space invasion; SCC: squamous cell carcinoma; AC: adenocarcinoma; FIGO: Federation International of Gynecology and Obstetrics. Quantitative variables are expressed as means ± standard deviation. P is derived from the Student’s t-test, chi-squared test or Fisher’s exact test between patients with and without LVSI.

### Radiomics feature extraction and selection

With PyRadiomics software, 1316 radiomics features were extracted from T2WI, DWI and DCE-T1WI-equilibrium phase images, respectively. We used mRMR, and LASSO to achieve further feature extraction, and finally, we obtained thirteen significant features. The details are in Table 3.

**Table 3:** Radiomics screening features

Sequence	Feature name	coefficient
DWI	wavelet-HLH_firstorder_Skewness(1)	-0.2413
DWI	wavelet-HHH_glszm_LowGrayLevelZoneEmphasis(2)	0.9184
DWI	lbp-3D-k_firstorder_Maximum (3)	1.0103
DWI	wavelet-HLL_glrlm_LowGrayLevelRunEmphasis(4)	1.2032
DWI	original_glszm_GrayLevelNonUniformityNormalized(5)	-1.9818
DWI	original_glszm_ZoneEntropy(6)	-2.1644
DCE-T1WI	wavelet-HHL_glcm_Correlation (7)	-0.3489
DCE-T1WI	lbp-3D-m2_firstorder_Variance (8)	0.2703
DCE-T1WI	wavelet-HHH_gldm_DependenceNonUniformityNormalized(9)	0.6023
T2WI	log-sigma-3-0-mm-3D_firstorder_Median (10)	0.4665
T2WI	original_shape_MajorAxisLength(11)	-0.1220
T2WI	wavelet-LHH_glrlm_RunVariance (12)	-0.0594
T2WI	wavelet-LHH_firstorder_Mean (13)	-0.5396

Note The larger the absolute value of characteristic coefficient is, the higher the prediction ability of LVSI is

### Performance of the MRI models

The MRI models showed a degree of prediction performance of LVSI status(Table 4). The predictive efficiency of the DWI model was higher than T2WI model and DCE-T1WI-equilibrium phase model in both training set and test set, but the mpMRI radiomics model provided an even better predictive model for LVSI, yielding an AUC of 0.835 and 0.817, and its accuracy, sensitivity, and specificity were higher than that of the single-sequence models. The mpMRI radiomics model is selected to construct the nomogram.

**Table 4:** Discriminative value of each parameter in differentiating LVSI

	AUC		Accuracy		Sensetivity		Specificity	
model	Training cohort	Testing cohort	Training cohort	Testing cohort	Training cohort	Testing cohort	Training cohort	Testing cohort
T2WI	0.671	0.668	0.65	0.648	0.585	0.5	0.724	0.808
DWI	0.77	0.758	0.707	0.707	0.615	0.536	0.81	0.846
DCE-T1WI	0.665	0.651	0.659	0.611	0.631	0.536	0.69	0.692
mpMRI radiomics	0.835	0.817	0.764	0.722	0.708	0.679	0.828	0.769
nomogram	0.838	0.837	0.78	0.722	0.846	0.786	0.707	0.654

Note AUC: area under ROC curve

### Performance of the radiomics nomogram

**Fig. 4:**
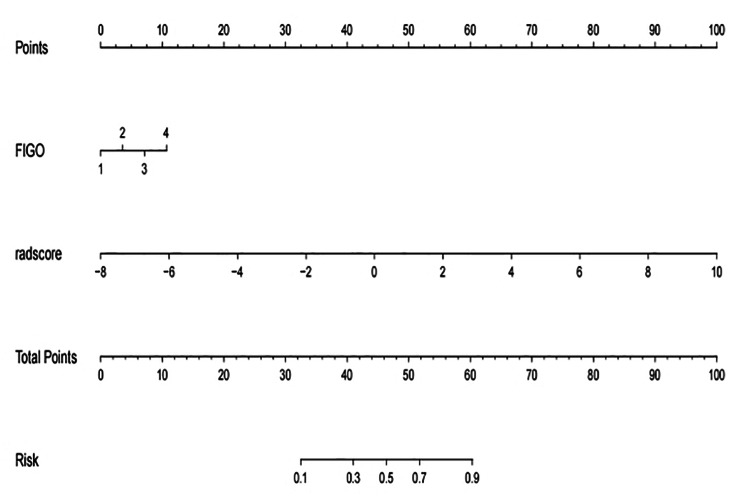
The radiomics-based nomogram

**Fig. 5:**
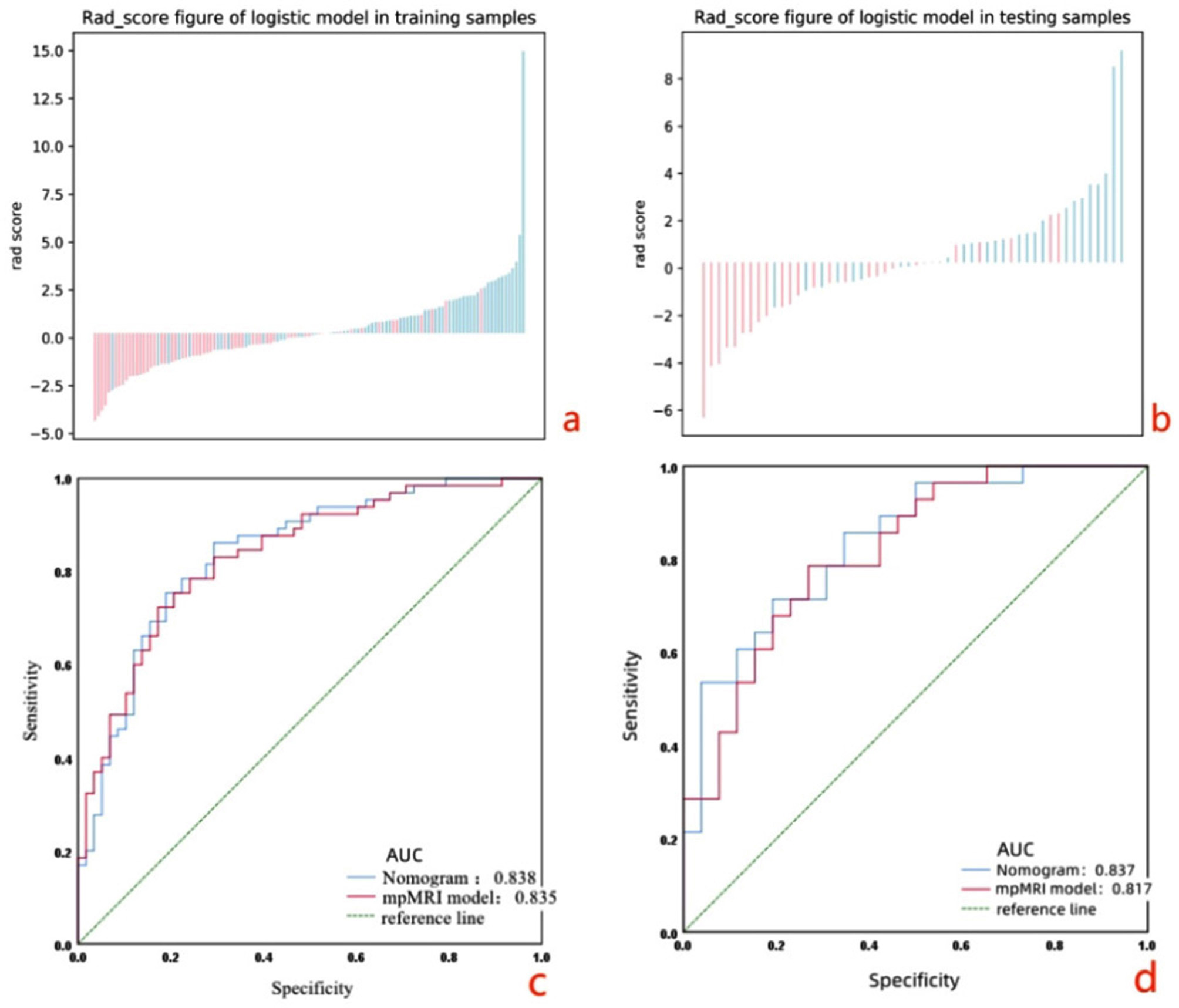
Rad-score of the mpMRI radiomics model in the training cohort (**a**) and the testing cohort; (**b**) the red bars represent the scores for patients without LVSI, while the blue bars represent the scores for those with LVSI. ROC curves of the nomogram and the mpMRI radiomics model in the training (**c**) and testing cohorts (**d**)

To provide clinicians with an easy-to-use tool, the FIGO stage and Rad-score were used to develop the nomogram in the training cohort (Table 5; Fig. 4). The AUC values of the nomogram for predict LVSI in the training and test cohorts were 0.838 (accuracy: 78.0%; sensitivity: 84.6%; specificity: 70.7%) and 0.837 (accuracy: 72.2%; sensitivity: 78.6%; specificity: 65.4%), respectively (Table 4; Fig. 5).

**Fig. 6:**
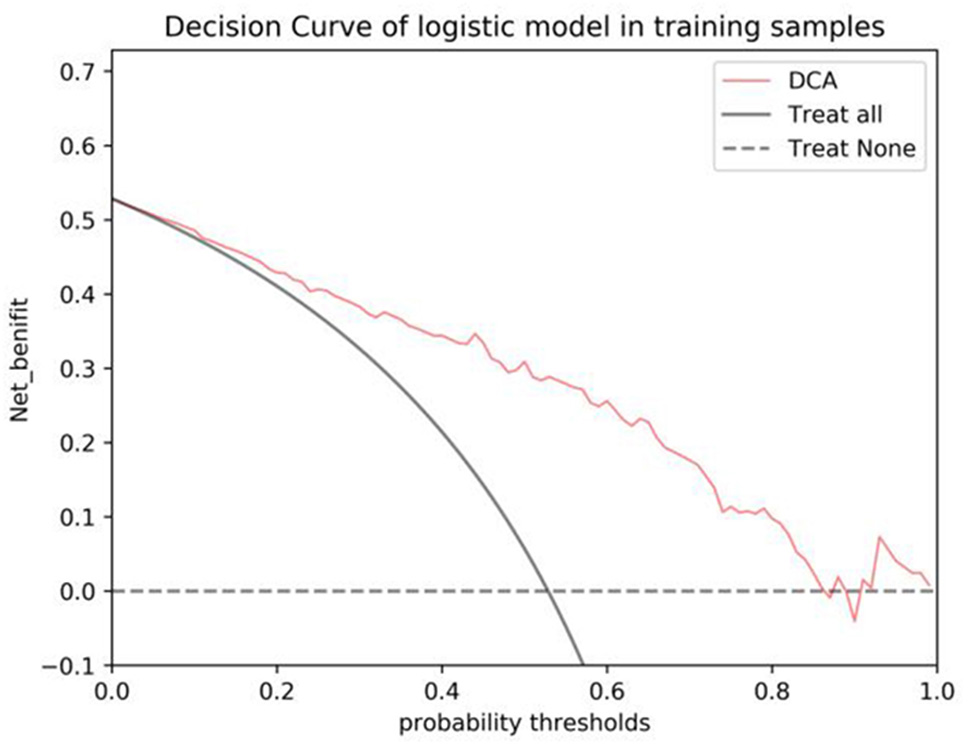
Decision curve analysis for the radiomics signature in the training cohort. The Y-axis shows the net benefit; the X-axis shows the threshold probability

**Fig. 7:**
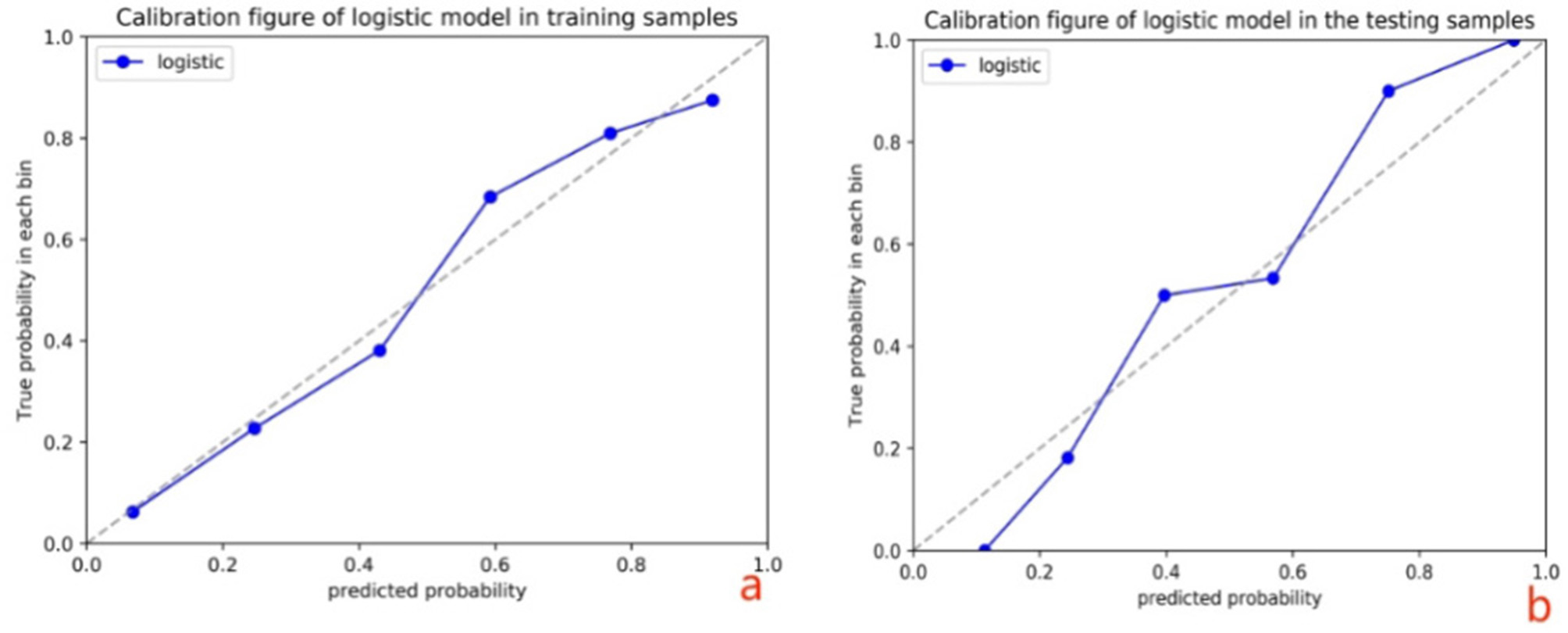
Calibration curves in the training cohort (**a**) and testing cohort (**b**). Closer fit to the diagonal line indicates a better evaluation

The DCA indicated that the nomogram was beneficial for predicting LVSI if the threshold probability of patients or doctors was from 0.01 to 0.87(Fig. 6). The calibration curve of the nomogram for the possibility of LVSI positive is shown in Fig. 7.

**Table 5:** Clinicopathological screening features

Parameters	Univariate logistic regression analysis		
	OR	95% CI	*P*
Age	0.983	(0.955, 1.012)	0.250
Histology	0.953	(0.402, 2.263)	0.914
Grade	0.849	(0.528, 1.364)	0.498
FIGO	1.746	(1.048, 2.909)	0.032*

Note *Significantly different (*p* < 0.050)

## Disscusion

At present, early CC (FIGO stage Ia-IIa) is mainly treated with surgical therapy, while intermediate and advanced CC often loses the chance of surgical radical treatment and is mostly treated with concurrent chemoradiotherapy(CCRT). Although chemoradiotherapy has improved the survival rate of advanced CC, there are still some patients with poor efficacy [6]. LVSI is closely associated with CC distant metastasis [13], and although it does not affect its clinical stage, it can affect the prognosis of patients and have an impact on clinical treatment decisions [6, 14]. In order to avoid delayed treatment, an accurate assessment of LVSI status can guide the clinical selection of drug types for CC patients, adjustment of radiation dose and CCRT plan, or surgery therapy. Currently, LVSI of CC can only be made through postoperative pathological evaluation. Conventional imaging examinations based on morphology cannot provide effective diagnostic information, and preoperative diagnosis is difficult. Therefore, it is of great significance to seek a non-invasive method for preoperative prediction of LVSI in patients with CC for clinical decision-making and patient prognosis.

In the retrospective research by Li et al. [15]. , it was shown that MRI images could be used as imaging predictive markers for LVSI. They developed and verified a radiomics model based on axial T1CE for preoperative LVSI prediction of CC, with AUCs of 0.754 and 0.727 in the training and testing sets, respectively. Du et al. [16]. combined radiomics features and clinical risk factors in developing a radiomics-based nomogram model, which achieved an AUC of 0.925 in the training cohort and 0.911 in the testing cohort. Also, in the current study, we found that the nomogram with the addition of clinical parameters has better diagnostic performance than the radiomics model alone. In Huang et al. [17]. ’s research, radiomics analysis of multiparametric MRI evaluates the presence of LVSI. They extracted the six most important features (3 from HR-T2WI, 1 from T2WI, 1 from FS-T2WI, and 1 from T1CE) to establish the predictive models. The AUCs in the training cohort and testing cohort were 0.922 and 0.940, respectively, which were significantly higher than the clinical models (AUCs were 0.709 and 0.730, respectively). These studies have verified the feasibility of MRI images in the preoperative prediction of LVSI status in patients with CC.

T2WI sequence can show higher contrast between the lesion and surrounding tissue. Among the 4 dominant features extracted by its single-sequence model, 2 belong to the first-order features, which indicates that the T2WI sequence can better show the voxel intensity distribution. The enhancement sequence is helpful to further explore the heterogeneity of the tumor because it shows the enhancement degree of the lesion and the contrast with the surrounding normal cervical wall after adding contrast agent. In this study, in order to further explore the predictive value of multiparametric MRI images for LVSI, a DWI sequence was added on the basis of conventional sequences. DWI is the most widely used MR functional imaging sequence. DWI uses diffusion (free movement) of water molecules in tissues to distinguish normal and diseased tissues and is able to reflect the cell density and cell size of tissues. DWI can reveal the characteristics of the tumor microenvironment, such as changes in extracellular matrix, vascular density, and oxygen supply, which are important factors affecting tumor growth and invasion. DWI is able to highlight the heterogeneity inside the tumor, which is an important feature in radiomics analysis [18]. The dominant features extracted from the single sequence DWI model are the most, 4 of which belong to gray texture features and 2 belong to first-order histogram features, which indicates that DWI sequence is more helpful to predict CC LVSI in terms of gray changes of tumors. The AUC value of the multi-sequence combined model was 0.835, which was significantly better than that of the single-sequence models. In order to establish a valuable prediction model for clinically assisted diagnosis, we drew a radiomics nomogram based on multi-sequence MRI combined with clinical features, which is a visualization model method widely used in the imaging field in recent years. The AUCs obtained in the training cohort and testing cohort were 0.838 and 0.837, respectively. In the training set, the accuracy, sensitivity and specificity were 0.78, 0.846 and 0.707, respectively, indicating that the model had low missed and misdiagnosed rates. A total of 13 features with the strongest predictive power were selected in this study, of which 7 were wavelet features, indicating that more dominant features came from the high-dimensional space that could not be recognized by naked eyes, which may be the main reason why even the high-definition MRI with the highest soft tissue resolution cannot accurately judge LVSI with the naked eye at present.

Different pathological types of tumors have different mechanisms and biological characteristics. Some studies [19] suggested that the positive rate of LVSI in cervical adenocarcinoma was lower than that in squamous carcinoma. However, the correlation between pathological types and LVSI was not found in this study, which may be related to the small sample size of cases. Accurate tumor stage and degree of differentiation are key factors for prognosis assessment and treatment decision making. At present, there are different viewpoints on the relationship between LVSI and the FIGO stage and differentiation degree. Pol et al. [4]. reported that LVSI was significantly correlated with the degree of differentiation, and the positive rate of LVSI was higher in patients with low differentiation than in patients with medium and high differentiation. Zhang et al. [20]. found no significant correlation between LVSI and differentiation degree and FIGO stage (P values were 0.099 and 0.398, respectively). The univariate analysis results of this study showed that LVSI was not correlated with the degree of differentiation, but was significantly positively correlated with FIGO stage, which was similar to the research results of Yan et al. [21]. At the same time, the radiomics nomogram combined with FIGO stage in this study also shows good predictive performance.

In 2018, FIGO updated the staging system of CC and proposed for the first time that pathological and imaging results were used for staging [6]. Clinical staging of CC was close to surgical and pathological staging. In this study, non-invasive methods were used to predict LVSI in patients with CC. Therefore, the 2018 FIGO staging based on imaging findings was used in this study, and all enrolled subjects were re-staged. Besides, there are some limitations to our study. First, all data in the present study were derived from the same institution and the sample size was small. In the future, an attempt will be made to conduct the multi-center study and expand the sample size. Second, manual segmentation of the ROIs is a labor-intensive and time-consuming process. It is important to promote the feasibility of radiometric measures to develop a reliable tool for the automatic segmentation and computation of radiomic signatures. Third, deep learning features based on convolutional neural networks are not incorporated in this study. Radiomics based on deep learning will also be a development trend in the future [22]. Fourth, genomic characteristics have not been incorporated in our nomogram. It is reported that DLL4 protein, COX-2 and TNC were closely related to LVSI of CC [23, 24]. An attempt will be made to explore the performance of adding this factor in our future research.

In conclusion, the radiomics nomogram model in this study based on mpMRI combined with clinical characteristic parameters has high diagnostic efficiency for preoperative prediction of LVSI in patients with CC, and further research is expected to verify and improve its predictive value.

### Electronic supplementary material

Below is the link to the electronic supplementary material.

Supplementary Material 1

Supplementary Material 2

## Data Availability

Data will be made available on request (m15350775172@163.com).
