# Supplementary figures and images for: Multiparametric mri-based radiomics nomogram for predicting lymph-vascular space invasion in cervical cancer
Source: BMC Med Imaging. 2024 Jul 5;24:167. doi: 10.1186/s12880-024-01344-y (PMC11225404; doi:10.1186/s12880-024-01344-y)

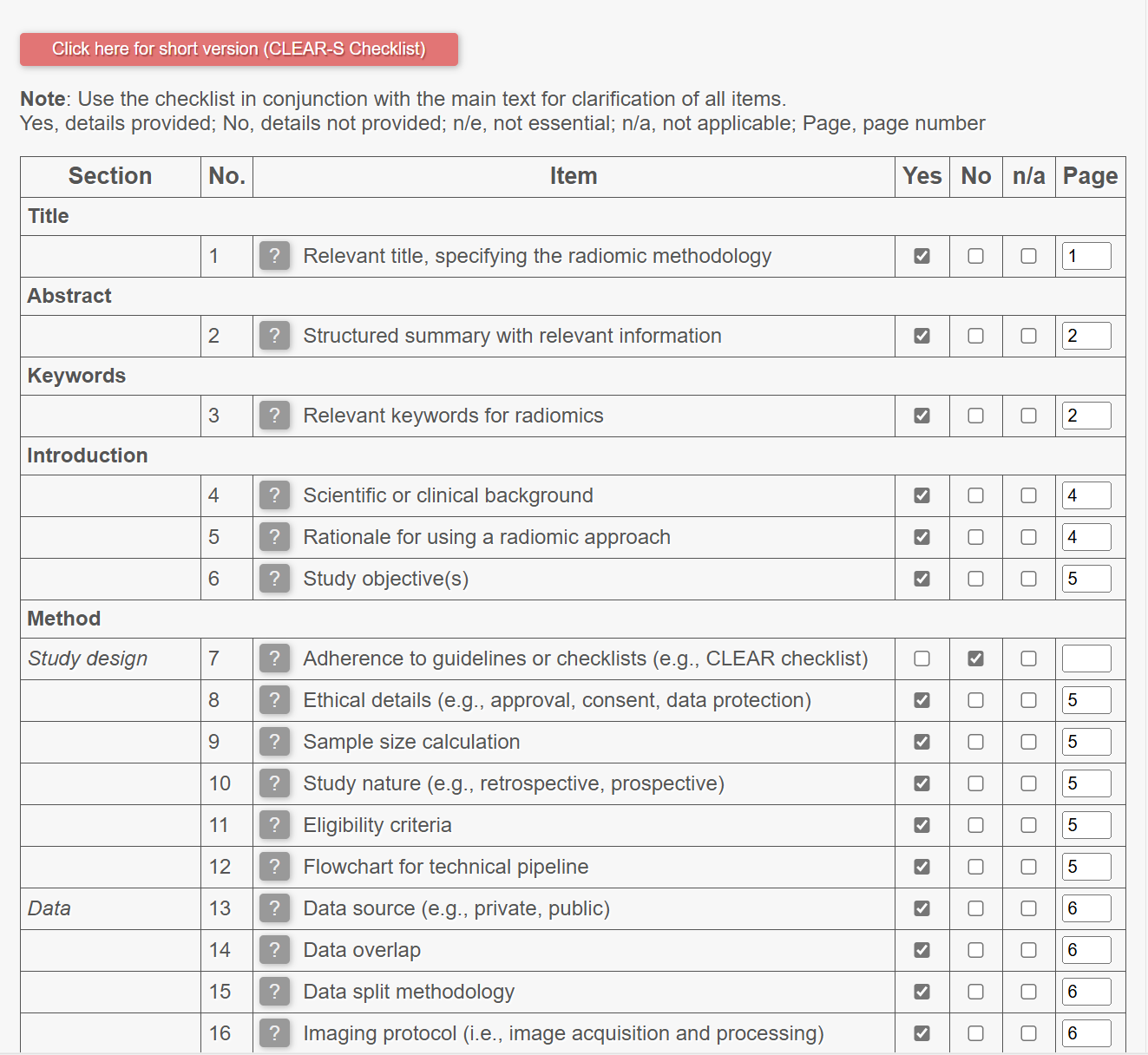

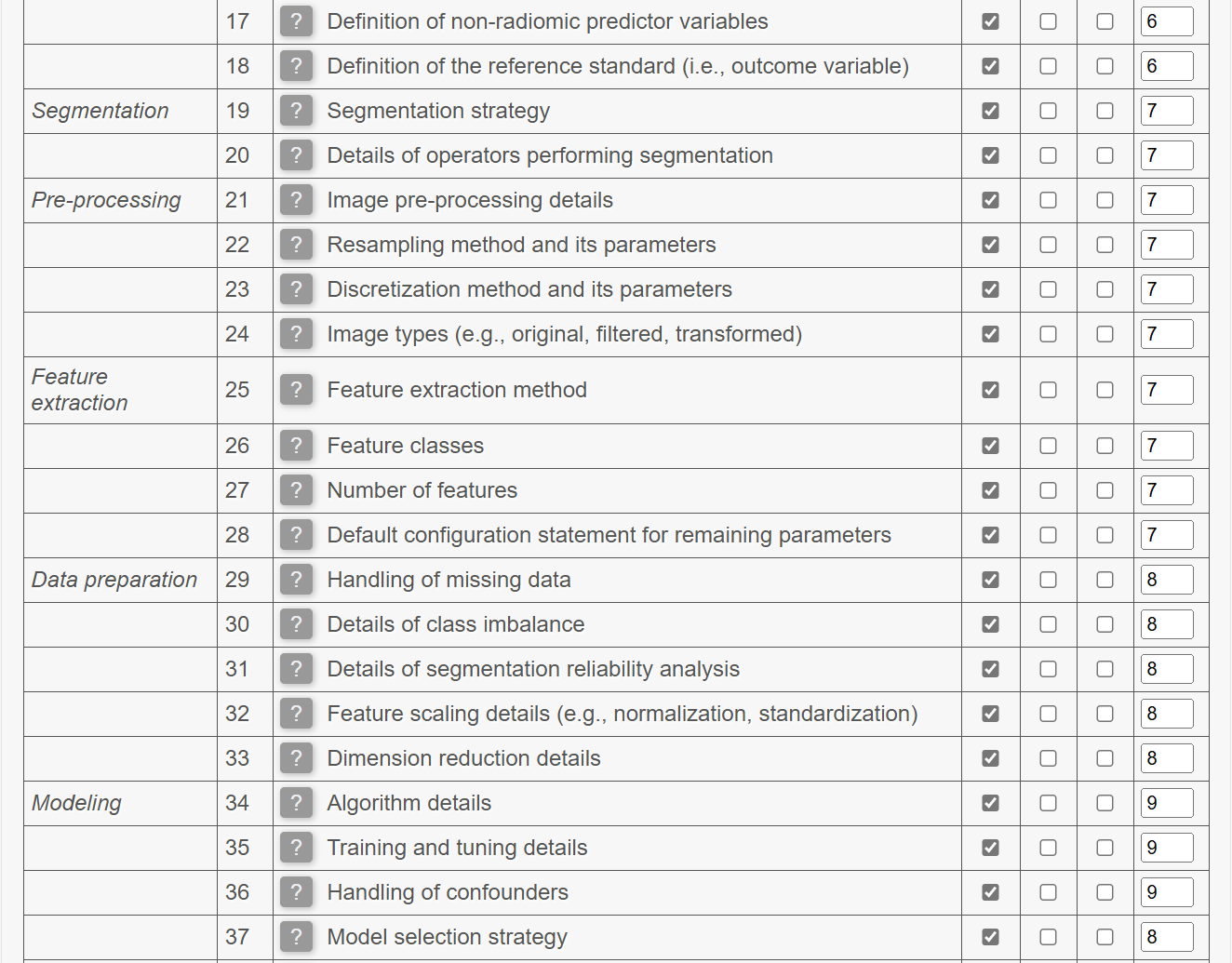

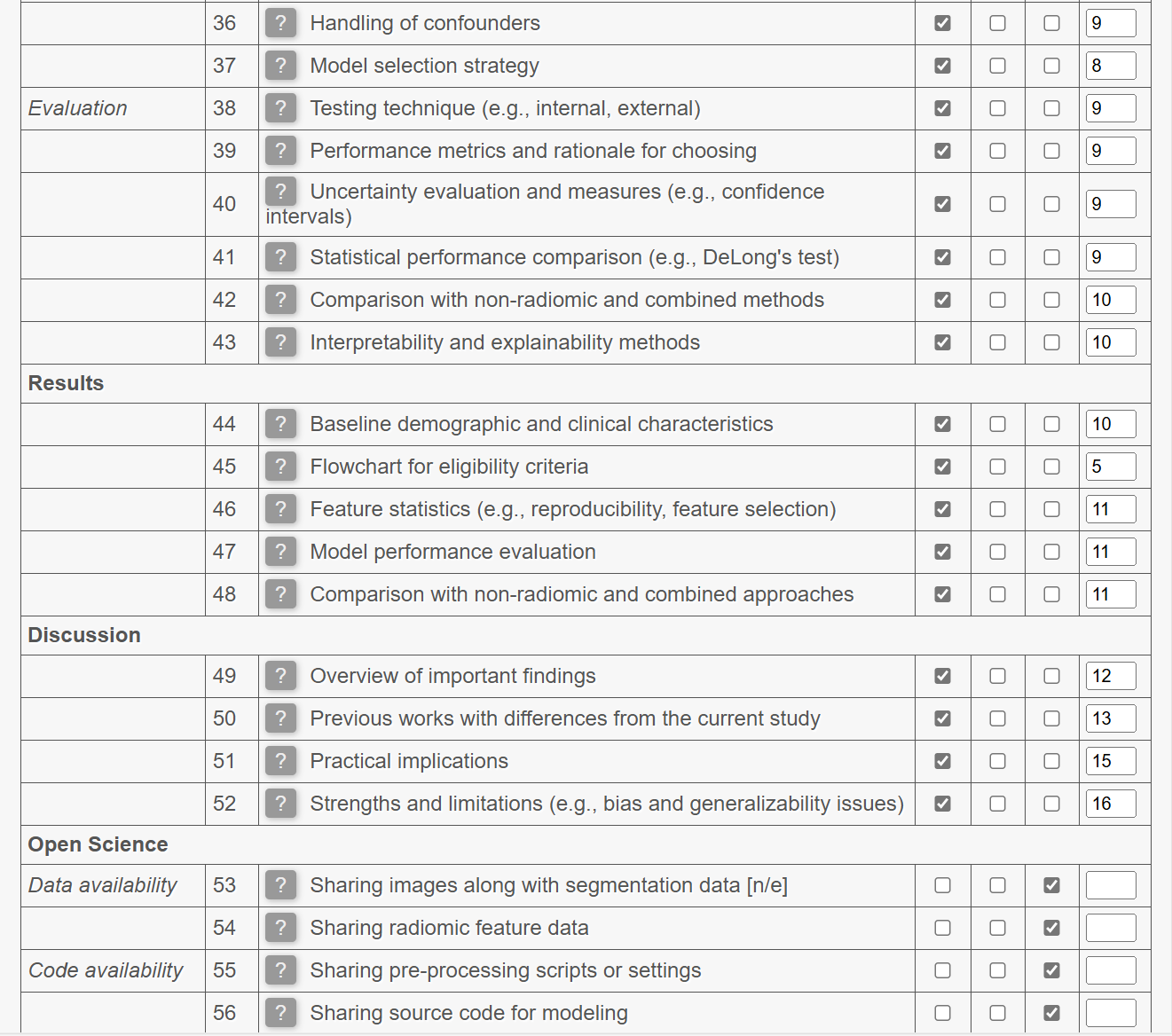

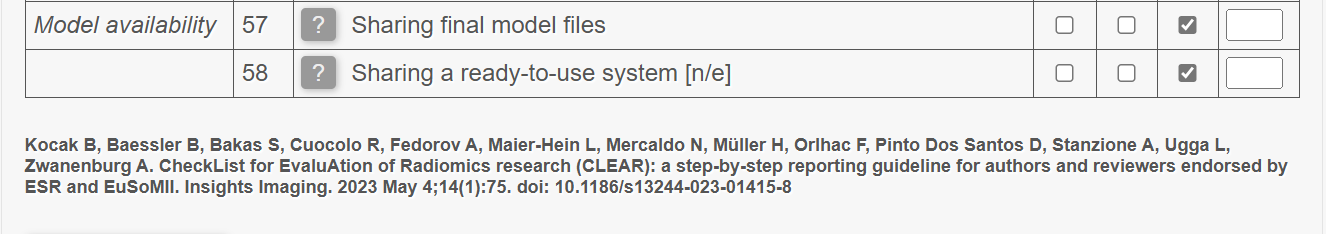

Supplement: Supplementary file 2 — Supplementary Material 2 [file 12880_2024_1344_MOESM2_ESM.docx]
